# Disseminated Fusariosis in a Patient With Acute Myeloid Leukemia: A Case Report

**DOI:** 10.7759/cureus.5922

**Published:** 2019-10-16

**Authors:** Navdeep Dehal, David Quimby

**Affiliations:** 1 Internal Medicine, Creighton University Medical Center, Omaha, USA; 2 Infectious Diseases, Creighton University Medical Center, Omaha, USA

**Keywords:** fusarium, fusariosis, aml, immunocompromised

## Abstract

We present a case report of disseminated fusariosis in a profoundly immunocompromised person. Early detection is key in this frequently-fatal infection; this case report emphasizes the findings with this infection and the need for chemoprophylaxis in appropriate patients.

## Introduction

As a ubiquitous environmental organism, Fusarium exposure is common but infection due to this organism is rare [1-3]. In the immunocompetent, infection is frequently localized and presents as keratitis or onychomycosis [1-2,4]. However, in the immunocompromised, disseminated disease is more common and frequently fatal [1-2,5-6]. Differentiating this from other opportunistic fungal infections is important, as this pathogen is often more resistant to antifungals than the most common fungal opportunist, Aspergillus [1-2,6]. The optimal antimicrobial therapy remains under debate.

## Case presentation

A 67-year-old woman with a past medical history of hypertension, valvular heart disease, and dyslipidemia presented with fatigue, dyspnea, decreased appetite, and jaundice. There was laboratory evidence of hemolytic anemia and thrombocytopenia. A bone marrow biopsy was consistent with acute myeloid leukemia (AML) with myelodysplastic changes. She started induction chemotherapy with a standard 7+3 regimen of cytarabine and idarubicin. During this induction phase, she was on antimicrobial prophylaxis with posaconazole, levofloxacin, and acyclovir. She tolerated this induction chemotherapy course fairly well, and her day 14 bone marrow showed no evidence of residual malignancy. She did develop neutropenic fever and was found to have bacteremia with Streptococcus pneumoniae, which was levofloxacin-sensitive (despite her being on this agent for chemoprophylaxis). She responded to empiric cefepime with de-escalation to oral cefuroxime as her neutrophil count recovered. She was dismissed from the hospital on only acyclovir prophylaxis.

A follow-up outpatient bone marrow showed ongoing remission of the AML, and she underwent monthly consolidation therapy with cytarabine; cycle three was delayed by two weeks due to leukopenia and thrombocytopenia. She declined bone marrow transplantation.

**Figure 1:**
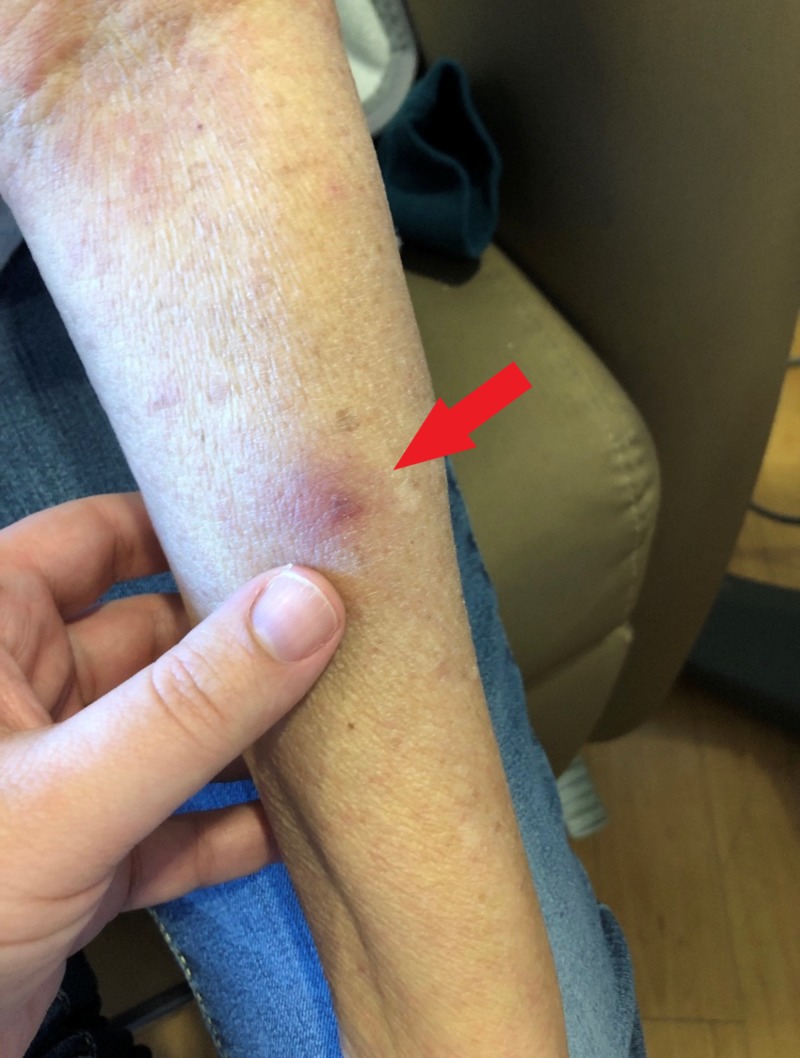
Arm lesion: painless subcutaneous nodule

**Figure 2:**
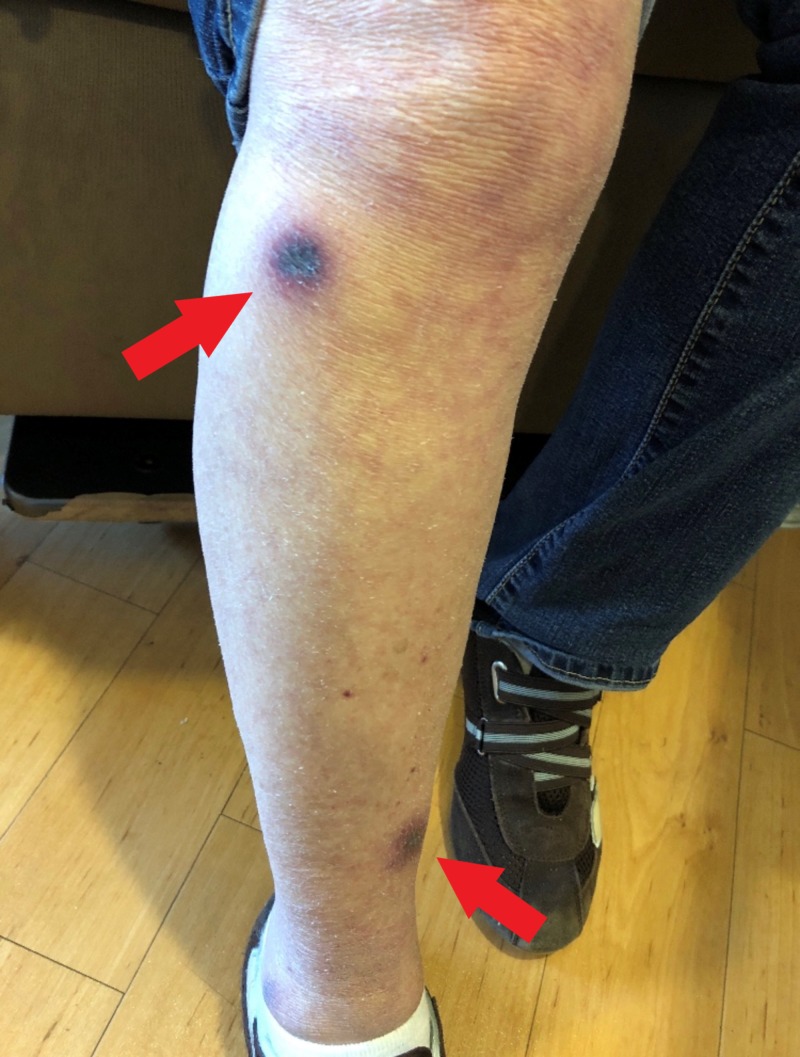
Leg lesions: flat, painless, ecchymotic areas with palpable subcutaneous nodules

Nine months after diagnosis, she was found to have a relapse of the AML. Therapy was started with decitabine, idarubicin, and cytarabine. She developed profound neutropenia. Her antimicrobial prophylaxis at this time consisted of trimethoprim-sulfamethoxazole, levofloxacin, and acyclovir; she was not on any antifungal prophylaxis during this period. Per review of the hematology-oncology records, it is unclear as to the reason for no antifungal prophylaxis; she had not previously had any adverse reactions to antifungal medication. Two weeks after starting this therapy, she noted some painless, nonpruritic, erythematous, slightly raised lesions on her arms and legs, less than 1 cm in diameter. As time passed, these remained asymptomatic but became darker in color and grew in size. These were noted in her medical record, but a biopsy was not offered at that time. Approximately four weeks after starting her chemotherapy, she developed increased fatigue, had more skin lesions (similar in character to the first lesions) forming on her trunk and all extremities, and noted that she was having asymptomatic fevers. She was admitted to the hospital. A physical examination revealed a total of eleven painless, nodular skin lesions on all extremities and her trunk; photographs were taken of characteristic lesions (Figures 1-2).

**Figure 3:**
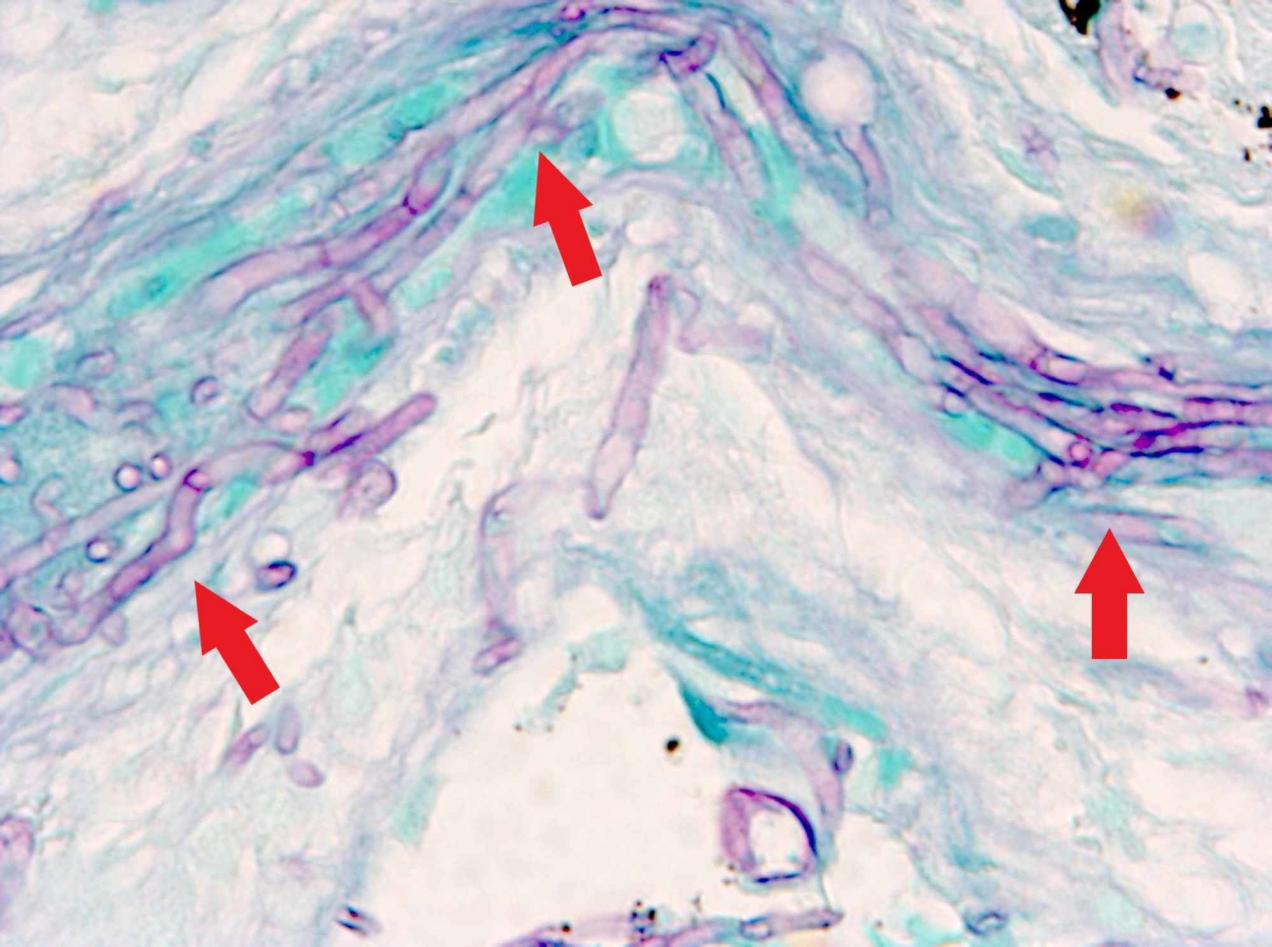
Right lower leg skin biopsy; periodic acid-Schiff (PAS) stain, 400x magnification showing dermal/epidermal necrosis and fungal hyphae

Laboratory studies showed a total white blood cell (WBC) count of 100 cells/mcl. Blood cultures were drawn and, given the skin lesions noted above, she was started on empiric cefepime for possible ecthyma gangrenosum and liposomal amphotericin B for fungal infection. Blood Aspergillus galactomannan was 0.21 (upper limit of normal 0.49), (1,3)-beta-D-glucan value was 263 pg/ml (normal < 80). A skin biopsy of the superior right lower leg lesion was performed (Figure 3).

With the biopsy showing fungal elements, voriconazole was added to her antibiotic regimen to better cover Aspergillus and Fusarium. She developed some visual hallucinations with the addition of the voriconazole, but these did subside. She defervesced within 24 hours of the addition of voriconazole. One of two routine aerobic blood cultures became positive at four days of incubation for what was initially called a yeast; this was identified as Fusarium species based on morphology. Skin biopsy culture grew the same organism. The isolate was sent to a reference laboratory (ARUP Laboratories, Salt Lake City, UT) for sensitivity testing and identified as Fusarium fujikuroi complex via matrix-assisted laser desorption/ionization-time of flight (MALDI-TOF); the sensitivity testing yielded an amphotericin B minimum inhibitory concentration (MIC) > 8 mcg/ml, itraconazole MIC > 16 mcg/ml, posaconazole MIC 0.5 mcg/ml, and voriconazole MIC 2 mcg/ml. Follow-up fungal blood cultures, drawn on day five of therapy, were eventually negative. Although offered, our patient declined CT imaging of the chest and sinuses to help fully determine the extent of infection.

She elected to stop active treatment, transitioned to hospice care, and passed away two weeks after hospital admission.

## Discussion

Fusarium species are common environmental fungi, capable of causing infections in both animals and plants [1-3]. In immunocompetent individuals, the most common forms of infection are keratitis (often associated with contact lens use) and onychomycosis [1-2,4].

More significant disease usually results from localized trauma (including burns) for invasive skin infection or inhalation of conida leading to pneumonia, sinusitis, or disseminated disease in the immunocompromised, consistent with the presentation in our case [1-3,5]. In one large literature review, 60/97 cases of invasive fusariosis were associated with hematologic malignancy; 11/97 cases had solid organ transplant as a risk factor [5].

**Figure 4:**
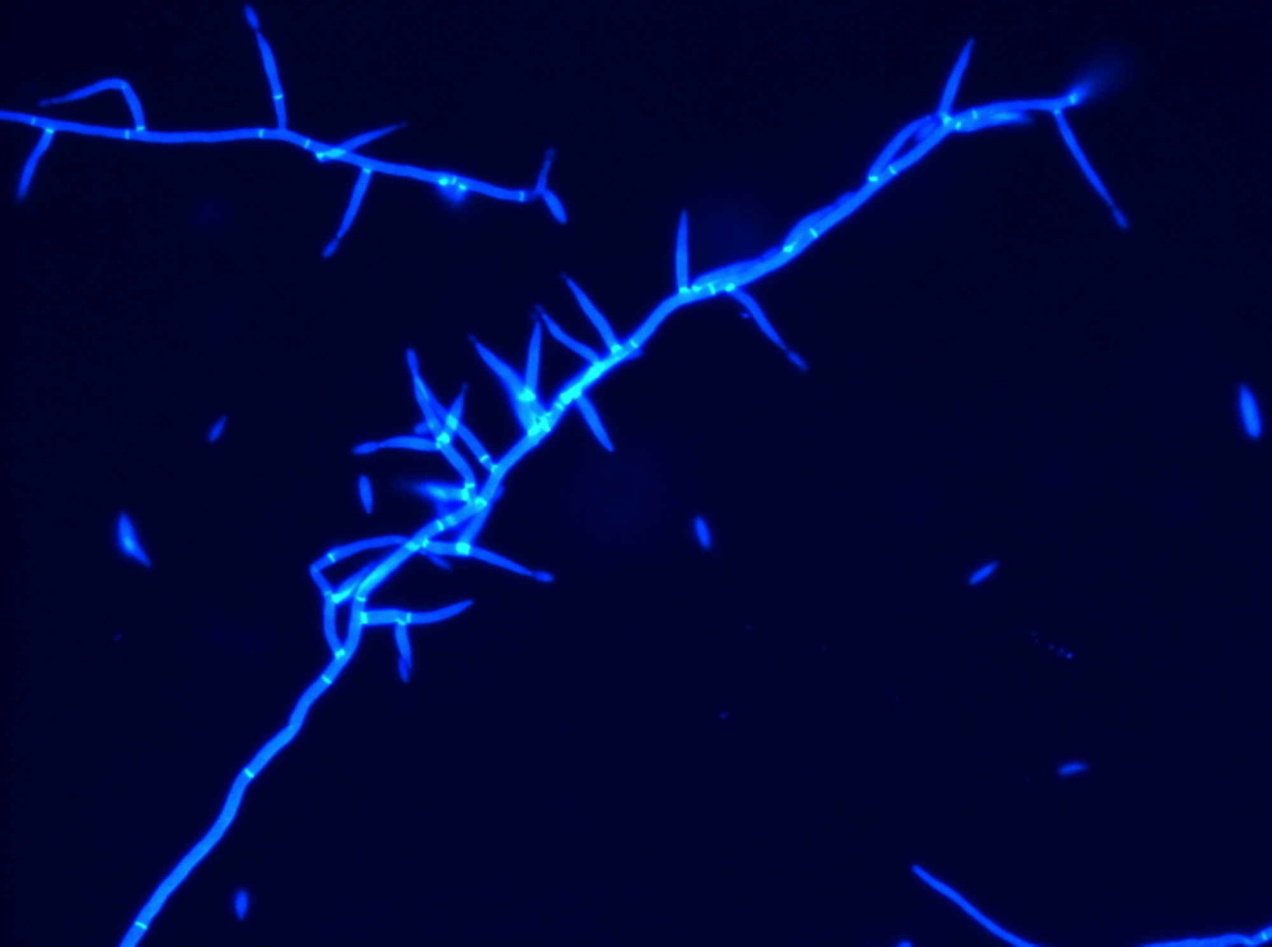
Tissue culture showing the branching hyphae of Fusarium; Calcofluor white stain, 400x total magnification

**Figure 5:**
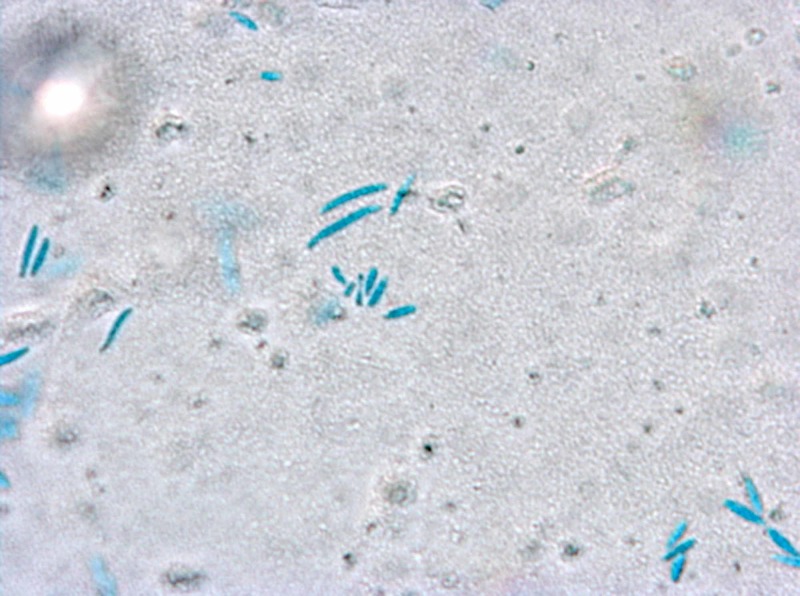
Macroconidia of Fusarium; aerobic blood culture stained with Lactophenol cotton blue dye, 400x total magnification

Fusarium is the second most common invasive fungal infection in the severely immunocompromised (behind Aspergillus)[1-2,6], so differentiation between the two is critical. Microscopically, Fusarium may resemble Aspergillus spp. with septate hyphae branching acutely or at right angles (Figure 4) [1-2,4]. However, the Fusarium genus may be characterized by the presence of “banana-shaped” macroconidia which can aid in identification, as we were able to demonstrate with Lactophenol cotton blue stain^ ^(Figure 5) [1-2,4]. In addition, Fusarium spp. are much more likely to grow in blood cultures than Aspergillus spp. (a significant percentage of cases of invasive or disseminated disease with Fusarium have positive blood cultures) [2-3,5-6], also helping differentiate the two before definitive identification can be made.

Fusarium tends to be more resistant to antifungal medications than Aspergillus. There are some in vitro data suggesting synergy between various antifungal agents [7-10], and while the optimal therapy for disseminated fusariosis is not clear, there are some guidelines [3] and dual antifungal agents are often used [11]. With this knowledge, our patient was treated with both liposomal amphotericin B and voriconazole and did clear her bloodstream infection, as shown by the fungal blood cultures obtained later in her hospital stay. Despite therapy, however, there remains a high mortality rate. Typically, in patients felt to be at risk for mold infections, fungal prophylaxis, usually directed against Aspergillus species, is warranted [12].

## Conclusions

The patient presented here is a fairly characteristic picture of disseminated Fusarium infection. She had a hematologic malignancy and had a prolonged, profound neutropenia. She then developed skin lesions consistent with disseminated fungal infection as well as fevers. Blood cultures were positive (typical for Fusarium infections, less so for Aspergillus infections), (1,3)-beta-D-glucan level was elevated, and blood Aspergillus galactomannan assay was negative, leading to suspicion of Fusarium (rather than Aspergillus) infection prior to pathology results and identification of the organism. Cases such as this one show the necessity of antifungal prophylaxis in patients with profound, protracted neutropenia. Although guidelines are clear on this need, fungal prophylaxis may be overlooked, as it was in this patient. This case also shows the need for evaluation of concerning skin findings in immunocompromised individuals; our patient first presented with skin lesions two weeks prior to eventual admission to the hospital. Treatment of disseminated Fusarium infections remains challenging, with optimal antifungal regimens not yet delineated.
